# Cost-effectiveness of on-demand removal of syndesmotic screws

**DOI:** 10.1007/s00068-022-02158-9

**Published:** 2022-11-14

**Authors:** D. Penning, F. R. K. Sanders, S. van Dieren, G. R. Roukema, J. Vermeulen, J. Winkelhagen, J. C. Goslings, T. Schepers, RODEO collaborator group, M. P. van den Bekerom, B. van Dijkman, J. A. Halm, J. M. Hoogendoorn, M. Parkkinen, R. N. van Veen

**Affiliations:** 1 Amsterdam UMC Location University of Amsterdam, Trauma Unit , Room J1A-214, Meibergdreef 9, 1105 AZ Amsterdam, The Netherlands; 2 Department of Epidemiology, Amsterdam UMC Location University of Amsterdam, Amsterdam, The Netherlands; 3 Surgery Department, Maasstad Hospital, Rotterdam, The Netherlands; 4 Department of Surgery, Spaarne Hospital, Hoofddorp, The Netherlands; 5 Department of Surgery, Dijklander Hospital, Hoorn, The Netherlands; 6 Department of Surgery, OLVG, Amsterdam, The Netherlands; 7 Department of Orthopedic Surgery, OLVG, Amsterdam, The Netherlands; 8 Department of Surgery, Flevo Hospital, Almere, The Netherlands; 9 Department of Surgery, Haaglanden MC, The Hague, The Netherlands; 10 Department of Orthopaedics and Traumatology, Helsinki University Hospital, Helsinki, Finland

**Keywords:** Ankle fractures surgery, Cost–Benefit analysis, Fracture fixation, internal/economics, Fracture fixation, internal/instrumentation, Implant removal, Quality of life

## Abstract

**Purpose:**

Syndesmotic screw removal following acute syndesmotic injury is a commonly performed procedure. However, recent studies suggest that the removal does not result in improved patient reported outcome, while the procedure has proved not to be without complications. The aim of this study was to present a health-economic evaluation of on-demand removal (ODR) compared to routine removal (RR) of the syndesmotic screw.

**Methods:**

Data were collected from the RODEO trial, a randomized controlled non-inferiority trial comparing functional outcome of ODR with RR. Economic evaluation resulted in total costs, costs (in Euro) per quality adjusted life year (QALY) and costs per point improvement on the Olerud Molander Ankle Score (OMAS). This included both direct and indirect costs.

**Results:**

Total costs for ODR were significantly lower with a mean difference of 3160 euro compared to RR (*p* < 0.001). The difference in QALY was not significant. The difference in OMAS at 12 months was 1.79 with an incremental cost-effectiveness ratio (ICER) of €-1763 (*p* = 0.512). The ICER was well below the willingness to pay. Although unit costs might vary between hospitals and countries, these results provide relevant data of cost-effectiveness.

**Conclusion:**

The clinical effectiveness of both ODR and RR can be considered equal. The costs are lower for patients treated with ODR, which leads to the conclusion that ODR is cost-effective.

**Supplementary Information:**

The online version contains supplementary material available at 10.1007/s00068-022-02158-9.

## Introduction

Syndesmotic screw removal is a commonly performed procedure within the field of orthopaedic trauma surgery. Syndesmotic screws are placed in order to fixate syndesmotic injuries in unstable ankle injuries, either with or without a fracture [1–3]. Although other fixation methods for syndesmotic injury (e.g. suture-buttons, bioabsorbable screws) are being used increasingly, the metallic syndesmotic screw thus far remains the most commonly used device [1–4]. The syndesmotic screw is a rigid fixation method, inhibiting motion of the distal tibiofibular joint. Combined with the risk of breakage, the assumed limitation in range of motion is why the screw is often removed after the syndesmosis has healed (8–12 weeks) [5, 6]. However, recent studies have suggested that the removal of this screw does not necessarily result in improved patient reported outcome [7–9]. Moreover, removing syndesmotic screws has proved not to be without complications, such as surgical site infections (SSI) [10].

Surgical site infections (SSI) occur frequently following implant removal [11]. SSIs are not only limiting functional recovery but are also associated with an increase in health care costs of up to €10,000 per patient [12–14]. By leaving the screw in place, these complications could be adverted, potentially resulting in improved overall outcome and in reduced health care costs. Syndesmotic screw removal may still be indicated in some cases, for example if the screw causes skin irritation, limited range of motion without screw loosening, or when there is a malposition of the fibula in the fibular notch of the tibia [8, 15]. If the syndesmotic screws were to be removed “on demand” (only in case of complaints), many procedures may be avoided. A recent randomized controlled trial (RCT) by Sanders et al. showed that on-demand removal (ODR) was non-inferior to routine removal (RR) of the syndesmotic screw when it comes to functional outcome [15]. The aim of the current study was to present a health-economic evaluation of ODR compared to RR of the syndesmotic screw in patients with acute syndesmotic injury. We hypothesize that there will be lower costs following ODR and therefore that ODR is cost-effective.

## Materials and methods

Data were collected from the RODEO trial, a randomized controlled non-inferiority trial comparing on-demand removal to routine removal of the syndesmotic screw, of which the full study protocol has been previously reported [16]. In summary, all adult (> 17 y/o) patients with acute syndesmotic injury (with or without accompanying ankle fracture), fixated within 14 days of trauma using one or two syndesmotic screws, were eligible for inclusion. Excluded were patients with an ISS score > 15, insufficient physical condition (to allow for potential removal surgery), concomitant injury of the ipsi- or contralateral ankle or other medical conditions hampering rehabilitation, and insufficient comprehension of Dutch, Finnish, Swedish or English language. Patients were included from 17 centres within the Netherlands and Finland, of which three were academic and 14 were teaching hospitals. As further elaborated on in the protocol, patients randomized for routine removal underwent removal of the syndesmotic screw 8–12 weeks after fixation, whereas in the ODR group, patients retained their screw unless they had complaints warranting removal [16]. The reasons for removals in the ODR group were pain, limited ROM, stiffness, revision surgery where new syndesmotic fixation was indicated, skin reaction to implants, screw backing out or patient’s wish not otherwise described. The primary outcome of this trial was functional outcome, measured by the Olerud Molander Ankle Score (OMAS). Secondary outcomes were the AOFAS (American Orthopaedic Foot and Ankle Society Ankle-Hindfoot scale), range of motion, quality of life (EQ-5D-5L), cost-utility and cost-effectiveness. Outcomes were all measured at 3, 6 and 12 months after syndesmotic fixation.

The trial was registered in the Netherlands Trial Register (NTR5965) and Clinicaltrials.gov (NCT02896998) and sponsored by ZonMw, a Dutch governmental organization (grant number: 843002705).

### Economic evaluation

The economic evaluation consisted of a cost-utility analysis (CUA) and a cost-effectiveness analysis (CEA) from a societal perspective with the costs per quality adjusted life year (QALY) and the costs per point functional outcome improvement as the primary economic outcomes. The costs were also compared to the willingness to pay. Both direct and indirect costs were used, based on average unit costs of 2018. The analyses were based on the intention-to-treat groups, using a time horizon of 12 months, since relevant differences in outcome and health care costs were expected to occur within that period.

### Resource utilization

Data on resource utilization were collected through questionnaires at 3, 6 and 12 months after syndesmotic fixation. The questionnaires used to evaluate in and out of hospital resource use were the iMTA Medical Consumption Questionnaire (iMCQ) and the iMTA Productivity Cost Questionnaire (iPCQ). In addition to this, patients’ medical records were screened after follow-up completion (12 months) to check for complications and revisions procedures. Resources were classified as either direct medical, direct non-medical or indirect non-medical costs. Direct medical costs were further subdivided into (1) regular in hospital costs, related to the index admission (syndesmotic screw removal, visits to outpatient clinic), (2) additional in hospital costs, related to complications or readmissions (additional implant removal, revision surgery, surgical debridement, readmission surgical ward, emergency room visits and ambulance rides), (3) out of hospital (para-)medical costs (general practitioner, company doctor, physiotherapist, home care). Indirect non-medical costs resulting from work absence or decreased productivity were determined using the iPCQ. To estimate loss of productivity, the friction costs method was used. Total costs per patient and per group were calculated by multiplying resources by the associated unit costs.

### Definition of outcomes

Quality of life was measured by the single value calculated from the EQ-5D-5L questionnaire on each time point (3, 6 and 12 months after syndesmotic fixation), using the standard Dutch tariff values[17]. Quality Adjusted Life Years (QALYs) were then calculated by multiplying the overall quality of life at each time point with the period of time, using the area under the curve method. Incremental cost-effectiveness ratios were calculated both as the difference in costs per QALY gained and as the difference in costs per additional point of improvement in functional outcome. Functional outcome was defined as the mean OMAS at 12 months, for which the difference between the two groups was calculated.

### Unit costs (in euro)

Costs of the various resources were extracted from the Dutch guideline on unit costs for health care, which was based on average health care prices in 2014[18][18]. To adjust these costs to the situation during the inclusion period of this study (2017–2019), a conversion factor of 1,041, based on the Consumer Price Index, was used in order to adjust all costs to the year 2018 [19]. Costs of specific procedures were based on DBC Health care products which are data of the Dutch Health Care Authority indicating costs for procedures based on insurance billing coledes, taking the 2018 price for each procedure [20]. Costs and units for each variable, adjusted to 2018, are displayed in Table 1 of the supplementary content.

**Table 1:** Baseline characteristics

	Routine Removal (*N* = 93)	On-Demand Removal (*N* = 104)
Sex, men	57 (61.3%)	62 (59.6%)
Age, mean (SD)	44.8 (1.6)	47.1 (1.4)
< 60 y/o	− 76 (81.7%)	− 84 (80.8%)
> / = 60 y/o	− 17 (18.3%)	− 20 (19.2%)
BMI, mean (SD)	28.1 (0.6)	28.3 (0.6)
*Missing*	*3*	*5*
Nicotine use	24 (27.9%)	25 (25.5%)
*Missing*	*7*	*6*
Alcohol abuse (> 2units/day)	7 (8.1%)	11 (11.2%)
*Missing*	*7*	*6*
Injury		
Weber B	26 (28.3%)	21 (20.2%)
Weber C	46 (50.0%)	54 (51.9%)
Maisonneuve	16 (17.4%)	26 (25.0%)
Isolated*	3 (3.3%)	2 (1.9%)
Other	1 (1.1%)	1 (1.0%)
* Missing*	*1*	
ASA classification		
I	46 (50.0%)	42 (41.2%)
II	43 (46.7%)	49 (48.0%)
III	3 (3.3%)	11 (10.8%)
* Missing*	*1*	*2*
No. Screw(s):		
1	63 (68.5%)	68 (66.0%)
2	29 (31.5%)	35 (34.0%)
* Missing*	*1*	*1*
Cortices:		
3	74 (80.4%)	84 (81.6%)
4	18 (19.6%)	19 (18.4%)
*Missing*	*1*	*1*

*ASA* American Society of Anesthesiologists, *BMI* body mass index (weight/(height^2^)), *No* number of, *y/o* years old

^*^Isolated syndesmotic injury (without fracture)

### Statistical analysis

Results are reported as means and mean difference along with their 95% confidence intervals and tested using independent sample t test. Differences in costs and effectiveness between the two groups were drawn and displayed graphically with cost-effectiveness planes. Incremental cost-utility (ICUR) and cost-effectiveness (ICER) ratios were calculated by dividing the mean difference in total costs per patient between treatment groups by the mean difference in QALYs and in OMAS, respectively. Therefore, ICER is the price in euro’s per point difference on the OMAS.

Missing data were handled according to the recent paper by Brand et al. [21]. Costs, QALYs and OMAS were multiple imputed at the item per time point level. Ten imputation sets were generated using MICE with predictive mean matching and combined using Rubins rule. Costs and effectiveness were predicted by randomized group, age, sex, BMI, smoking, alcohol, diabetes, chronic obstructive pulmonary disease, American Society of Anesthesiologists (ASA) classification, type of injury, all health care costs, productivity loss, QALYs and OMAS. Dependencies over time were taken into account by including measurements at all time points.

The confidence intervals of the ICER and the cost-effectiveness plane were based on bias-corrected and accelerated non-parametric bootstrapping of 1000 bootstrap samples after combining the multiple data sets into one mean single imputation [21].

In addition to the primary CUA and CEA analyses, preplanned subgroup-analyses were performed for patients younger/older than 60 years old and for patients with/without a SSI. Sensitivity analyses were performed by varying medical costs of screw removal, outpatient clinic visits, emergency room visits and revalidation clinic admissions by 30%. Analyses were performed using R version 3.6.1 (R Foundation for Statistical Computing, Vienna, Austria).

## Results

Between January 2017 and April 2019, 197 patients were included in the RODEO trial, of which 93 randomized for RR and 104 for ODR of the syndesmotic screw. Baseline characteristics of all patients incorporated in the current economic evaluation are shown in Table 1. Overall response rates of the questionnaires were 69.5%, 68.5% and 68.5% at 3, 6 and 12 months, respectively.

### Costs and resource use

Table 2 shows the difference in resource utilization and costs in total and as mean per patient for each group. There was a significant difference in total costs per patient (sum of direct and indirect costs) of − €3160 (95% CI − €4170 to − €2149, *p* < 0.001), in favour of ODR. This difference was made up by the difference in direct costs; 1. Costs for syndesmotic screw removal, which were significantly lower in the ODR group, as expected (− €1000, 95% CI − €1166 to − €835, *p* < 0.001), 2. More readmissions and surgical debridements in RR, leading to a mean difference per patient of -€1,274 (95% CI -€2,690 to €142, p = 0.077), and 3. A shorter duration of revalidation and home care in the ODR group (− €144, 95% CI − €341 to €53, *p* = 0.152). Also included in the difference were the indirect non-medical costs, which were €503,985 for RR and €482,891 for ODR (Table 2).

**Table 2:** Resource utilization and (mean) costs, as indexed for 2018

		Routine Removal (*n* = 93)		On-Demand Removal (*n* = 104)	
	Unit	Total units	Total costs (€)	Total units	Total costs (€)
Index admission					
Syndesmotic screw removal	Procedures	82	127,920	25	39,000
Mean subtotal per patient (95% CI)		1375 (1343–1408)		375 (334–416)	
Mean difference in subtotal (95% CI)		− 1000 (− 1166 to − 835, *p* < 0.001)			
Readmissions and reinterventions					
Removal of other material	Procedures	4	6,240	4	6240
Revision reconstruction /syndesmotic fixation	Procedures	9	31,050	9	31,050
Surgical debridement	Procedures	4	13,800	1	3,450
Readmission Surgical Ward	Days	120	50,593	80	33,729
Emergency room visits/ ambulance	Frequency	94	50,395	79	42,353
Revalidation	Days	148	70,871	0	0
Subtotal		222,949		116,822	
Mean subtotal per patient (95% CI)		2397 (1018 to 3776)		1123 (794–1,4527)	
Mean difference in subtotal (95% CI)		−1274 (− 2690 to 142, *p* = 0.077)			
Regular care					
Outpatient clinic visits Dep. of surgery	Visits	226	17,174	190	14,438
Physician		138	4,740	169	5805
Company doctor		198	19,996	198	19,996
Physiotherapist		1393	47,850	1420	48,777
Home care	Days	113	5316	87	2317
Subtotal		95,076		91,333	
Mean subtotal per patient (95% CI)		1022 (871–1174)		878 (750–1007)	
Mean difference in subtotal (95% CI)		− 144 (− 341 to 53, *p* = 0.152)			
Other costs					
Out of pocket expenses	Euro		2,508		2051
Total direct medical costs			448,453		249,207
Indirect non-medical costs			503,985		482,891
Total costs			952,438		732,098
Mean total costs per patient (95% CI)		9913 (9073–10,754)		6753 (6190–7316)	
Mean difference in total costs per patient (95% CI)		− 3,160 (− 4170 to –2149, *p* < 0.001)			

### Cost-utility

**Fig. 1:**
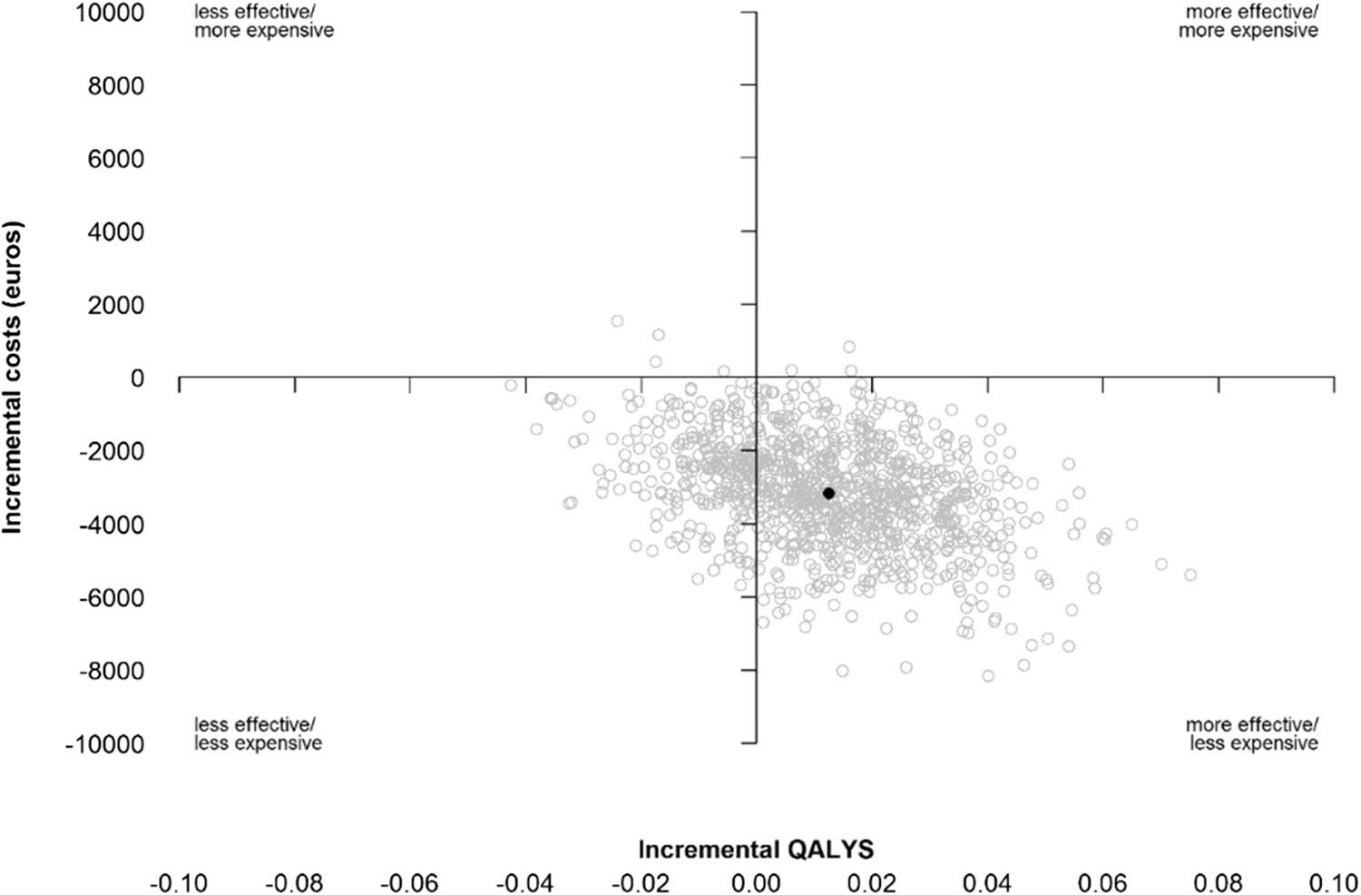
Effectiveness plane of QALYS compared to Incremental costs in Euros

The mean QALYs in 1 year was 0.795 (95% CI 0.727–0.862) in the group with on-demand removal and 0.782 (95% CI 0.753–0.811) in the group with routine removal, which lead to a difference in QALYs of 0.013 (95% CI − 0.026 to 0.051, *p* = 0.474). This difference was not statistically significant, nor clinically relevant. Figure 1 shows the cost-effectiveness plane for QALYS. The incremental cost-utility ratio was -€252,673 (95% BCa CI: − €24,219,700 to €65,306).

### Cost-effectiveness

**Fig. 2:**
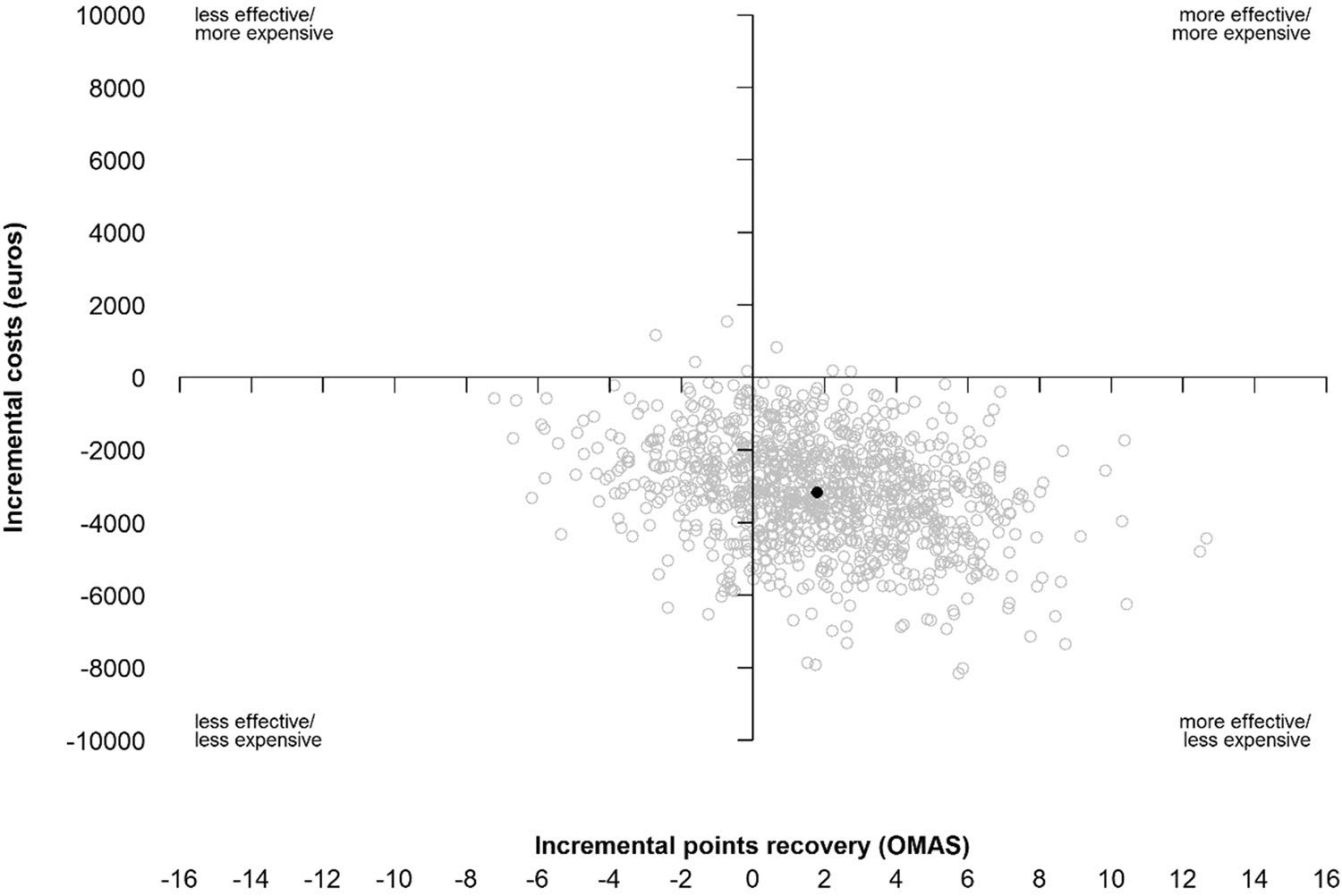
Effectiveness plane of functional outcome score in OMAS compared to Incremental costs in Euros

The mean difference in functional outcome, measured by the OMAS at 12 months, was 1.79 (95% CI − 4.92 to 8.50), with a mean OMAS of 78.62 (95% CI 66.90–90.34) in the ODR group and 76.83 (95% CI 71.82–81.83) in the RR group. The ICER was in favour of ODR: − €1.763 (95% CI − €564,687 to €166, *p* = 0.512). This is shown in Fig. 2.

### Subgroup analyses

The subgroup analysis based on age (< 60 vs. > / = 60) showed cost-effectiveness for ODR when it comes to functional outcome for patients under 60 y/o. For every point difference on the OMAS, there was a difference of − €3,333 (− €8,253,179 to − €1,097) between ODR and RR with less expenses in the ODR group (Table 2 of Supplementary content). ODR in patients < 60 costs €1,017,572 less per QALY and a QALY costs €43,716 less in patients ≥ 60 years.

The subgroup analysis which excludes patients with a SSI is specified in the supplementary content and had a mean difference in costs of €− 3226 (− €6822 to €370), mean difference in functional recovery by OMAS at 12 months of 1.34 (− 5.35 to 8.04) and a mean difference in QALY over 12 months of 0.014 (− 0.026 to 0.054). Costs, functional recovery and QALY were more favourable in the ODR group. The ICUR was -€230,542 (€− 219.210.538 to €54.613).

Following sensitivity analyses, medical costs all showed a difference in costs between RR and ODR, in favour of ODR (Table 3).

**Table 3:** Sensitivity analyses of medical costs per patient

	ODR (€)	RR (€)	Cost difference (€)
Total medical costs (base analysis)	2396 ( 2020–2840)	4822 (3901–7522)	− 2426 ( − 4916 to − 1437)
Syndesmotic screw removal			
-30%	1967 ( 1682–2345)	4055 ( 3224–7276)	− 2,088 ( − 5110 to − 1200)
+ 30%	2228 ( 1880–2649)	4921 ( 4075 to 8092)	− 2693 ( − 5473 to − 1765)
Outpatient visits			
-30%	2392 ( 2025–2839)	4804 ( 3879–7493)	− 2413 ( − 4895 to − 1432)
+ 30%	2507 ( 2126 to 2963)	4948 ( 4020–7627)	-2441 ( − 4887 to − 1444)
ER visits			
-30%	1975 ( 1665–2353)	4326 ( 3496–7516)	− 2350 ( − 5263 to − 1464)
+ 30%	2220 ( 1897–2634)	4651 ( 3787–7827)	− 2431 ( − 5240 to − 1497)
Removal of revalidation			
	2098 ( 1794–2508)	3726 ( 3323–4209)	− 1628 ( − 2234 to − 1088)

## Discussion

The mean difference in total costs was €3,160 per patient in favour of the ODR group. Even though the upper limit of the 95% confidence interval of the ICER was marginally higher than zero, it was well below the level of willingness to pay, which is 50.000 euro, which makes ODR a cost-effective intervention [22].

This is the first cost analysis study to focus solely on screw fixation for syndesmotic injury, comparing cost-effectiveness of syndesmotic screws removed routinely or on demand. Previously performed cost analyses have focussed on the cost-effectiveness of screw versus dynamic implants, such as the suture button [23–25]. Neary et al. described that when comparing a tightrope to not-routinely removed syndesmotic screws, less than 10% of screws would have to be removed in order to be cost-effective [23]. However, their study was based solely on numbers from the literature, and not on original data. Moreover, their calculations were based on a revision (removal) surgery price of $14,768, which is much higher than the €1560 ($1796 based on exchange rate of 2018) that was indexed in the Netherlands for this procedure in 2018. Weber et al. found that cost-effectiveness of screw and Suture button fixation were more dependent on the removal rate and that they would be equivalent in price when the removal rate of screws would be between 18 and 53%, assuming that the removal rate of Suture button is 0[25]. Since the removal rate in the ODR group in our trial was 23%, this would make it cost-effective or at least equivalent to use a syndesmotic screw. Although long-term complications of retaining syndesmotic screws have not been studied yet, several long-term complications of dynamic implants (e.g. infections, osteomyelitis, osteolysis, and diastasis as a result from failed stabilization) have been described, which have not been incorporated in any of the above-mentioned studies [26–29]. Combining these complications with the cost-effectiveness of ODR of syndesmotic screws makes a strong case for keeping/implementing ODR of the syndesmotic screw as standard treatment.

In this study, the direct costs were higher following RR, mainly because of the costs of higher frequency of surgery and more readmissions at the surgical ward. In addition, we expected the costs to be higher due to the higher rate of SSIs in the RR group, resulting in higher health care costs. Therefore, we also calculated the difference in costs excluding patients with SSI, which had no relevant effect on the results. Thus, the difference in costs cannot be explained solely by the amount of SSIs in the RR group.

For indirect costs, the iPCQ and the friction costs method with age-adjusted mean daily wages were used to estimate loss of productivity. Indirect costs, defined as lost hours of productivity, were higher in the RR group. Because this cohort was part of a RCT, we can assume that the hourly wage and therefore lost productivity costs were equally divided between both groups and that this does not account for the difference.

The functional outcome after 12 months, measured with OMAS, did show a difference of 1.79 with higher scores following ODR [15]. This leads to an ICER of €-1763 (the difference in costs per point at the OMAS), supporting the suggestion to opt for ODR.

In the subgroup analysis comparing patients aged < 60 years and patients ≥ 60 years, we found that the costs gained when choosing for ODR are higher in the younger group, which can be attributed to the fact that the younger group has less indirect costs following ODR because these costs include costs made by work deprivation. The results of this subgroup analysis could be taken into account in daily practice.

This study compared ODR and RR following syndesmotic injury, and the removal was performed at the operation room. Sugi et al. recently state that it is safe to remove the syndesmotic screw in a clinical setting out of the operating room based on a complication rate of 2 SSIs in 170 patients [30]. This might affect removal costs but is not likely to change OMAS and QALYs after 12 months compared to our study. Since these results are from one single study and costs may vary compared to our study, an additional RCT with clinical setting for removal might attribute to the current literature.

A limitation of our study was that unit costs might vary between hospitals due to overhead costs, which was not accounted for in the current study, leading to an estimation of the costs per patient and in total. However, due to the randomized set-up of the study, uncertainties are expected to be equally divided. Additionally, in this study, only costs made within 12 months after syndesmotic fixation were incorporated. The results thereby do not include removal of screws after this period of time, or potential late complications of retaining the syndesmotic screw. We know that if the retained syndesmotic screw breaks, the location of breakage affects the chance of cortical erosion due to the broken syndesmotic screw [31]. But to the best of our knowledge, there have not been any trials studying the long-term complications and outcome of retaining syndesmotic screws. Therefore, we are unable to say what the consequences for costs would be.

## Conclusion

The clinical effectiveness of both ODR and RR can be considered equal. The costs are lower for patients treated with ODR, which leads to the conclusion that ODR is cost-effective.

## Supplementary Information

Below is the link to the electronic supplementary material.

Supplementary file1 (DOCX 18 KB)

## Data Availability

The datasets analyzed during the current work are available upon reasonable request from the corresponding author.
