# Supplementary material for: Cost-effectiveness of on-demand removal of syndesmotic screws
Source: Eur J Trauma Emerg Surg. 2022 Nov 14;49(2):921–8. doi: 10.1007/s00068-022-02158-9 (PMC10175308; doi:10.1007/s00068-022-02158-9)
Supplement: Supplementary file 1 — Supplementary file1 (DOCX 18 KB) [file 68_2022_2158_MOESM1_ESM.docx]

**Supplementary content**

**Table 1. Costs and units for each variable**

|  | Unit | Costs (€)/unit |
| --- | --- | --- |
| Syndesmotic screw removal | Procedures | € 1560.- |
| Removal of other material | Procedures | € 1560.- |
| Revision reconstruction/ syndesmotic fixation | Procedures | € 3450.- |
| Surgical Debridement | Procedures | € 3450.- |
| Other treatment without readmission | Procedures | € 421.60 |
| Readmission surgical ward | Days | € 421.61 |
| Emergency room visits | Frequency | € 269.62 |
| Ambulance transport | Frequency | € 536.12 |
| Revalidation | Days | € 478.86 |
| Outpatient clinic visits   - Surgery - Other | Frequency | € 75.99 € 94.73 |
| (para-)medical care   - General practitioner - Company doctor - Physiotherapist - Home care   - Household   - Help   - Nurse | Frequency Frequency Frequency Days | € 34.35 € 196.- € 34.35  € 23.94  € 52.05 € 75.99 |

|  | <60 years | | | ≥60 years | | |
| --- | --- | --- | --- | --- | --- | --- |
|  | ODR  N=86 | RR  N=78 | difference | ODR n=18 | RR n=15 | difference |
| Costs | 7,365 (-637 to 15,368) | 10,595 (6,898 to 14,291) | -3,229 (-7,536 to 1,077) | 3,831  (-3,375 to 11,036) | 6,372  (3,637 to 9,108 | -2,542 (-7,012 to 1,928) |
| Qaly | 0.788 (0.711 to 0.864) | 0.785 (0.752 to 0.817) | 0.003 (-0.041 to 0.047) | 0.828 (0.693 to 0.962) | 0.770  (0.712 to 0.827) | 0.058 (-0.019 to 0.135) |
| ICUR |  |  | -1,017,572 (-3,576,276 to 833,726) |  |  | --43,716 (-484,781 to 3,908) |
| OMAS | 78.55 (66.03 to 91.08) | 77.58 (72.27 to 82.90) | 0.97 (-6.24 to 8.18) | 78.94 (51.69 to 106.2) | 72.90 (61.22 to 84.58) | 6.04 (-9.52 to 21.61) |
| ICER* |  |  | -3,333 (-8,253,179 to -1,097) |  |  | -420 (-73,896 to 338) |

**Table 2. Subgroup analysis for people below and above 60 years**

Subgroup Excluding SSI (6 in RR and 1 in ODR)

Mean difference in costs: -3,226 (-6,822 to 370)

Costs in ODR: 6,739 (5 to 13,472)

Costs in RR: 9,965 (6,827 to 13,103)

Mean difference in functional recovery by OMAS at 12 months 1.34 (-5.35 to 8.04)

- Mean OMAS at 12 months in ODR 78.66 (66.77 to 90.54)
- Mean OMAS at 12 months in RR 77.31 (72.12 to 82.50)

Mean difference in QALY over 12 months: 0.014 (-0.026 to 0.054)

- Mean QALY over 12 months in ODR 0.795 (0.726 to 0.865)
- Mean QALY over 12 months in RR 0.781 (0.752 to 0.811)

ICER QALY: -230,542 (-219.210.538 to 54.613)
